# Comparing the ‘When’ and the ‘Where’ of Electrocortical Activity in Patients with Tourette Syndrome, Body-Focused Repetitive Behaviors, and Obsessive Compulsive Disorder

**DOI:** 10.3390/jcm13092489

**Published:** 2024-04-24

**Authors:** Sarah Desfossés-Vallée, Julie B. Leclerc, Pierre Blanchet, Kieron P. O’Connor, Marc E. Lavoie

**Affiliations:** 1 Laboratoire de Psychophysiologie Cognitive et Sociale, Montréal, QC H1N 3J4, Canada; sarah.desfosses-vallee@umontreal.ca; 2 Centre de Recherche de l’Institut Universitaire en Santé Mentale de Montréal, Montréal, QC H1N 3J4, Canada; leclerc.julie@uqam.ca (J.B.L.); pierre.j.blanchet@umontreal.ca (P.B.); kieron.oconnor@umontreal.ca (K.P.O.); 3 Département de Psychologie, Université de Montréal, Montréal, QC H3C 3J7, Canada; 4 Département de Psychologie, Université du Québec à Montréal, Montréal, QC H2X 3P2, Canada; 5 Centre de Recherche CIUSSS du Nord-de-l’île-de-Montréal, Montréal, QC H4J 1C5, Canada; 6 Faculté de Médecine Dentaire, Département de Stomatologie, Université de Montréal, Montréal, QC H3C 3J7, Canada; 7 Département de Psychiatrie et Addictologie, Université de Montréal, Montréal, QC H3C 3J7, Canada; 8 Département de Sciences Humaines, Lettres et Communication, Université TÉLUQ, Quebec City, QC G1K 9H6, Canada

**Keywords:** Tourette Syndrome, obsessive compulsive disorder, body-focused repetitive behaviors, tics, event-related potentials, P200, N200, P300, sLORETA

## Abstract

**Background/Objectives**: Tourette Syndrome (TS), Obsessive Compulsive Disorder (OCD), and Body-Focused Repetitive Behaviors (BFRB) are three disorders that share many similarities in terms of phenomenology, neuroanatomy, and functionality. However, despite the literature pointing toward a plausible spectrum of these disorders, only a few studies have compared them. Studying the neurocognitive processes using Event-Related Potentials (ERPs) offers the advantage of assessing brain activity with excellent temporal resolution. The ERP components can then reflect specific processes known to be potentially affected by these disorders. Our first goal is to characterize ‘when’ in the processing stream group differences are the most prominent. The second goal is to identify ‘where’ in the brain the group discrepancies could be. **Methods**: Participants with TS (*n* = 24), OCD (*n* = 18), and BFRB (*n* = 16) were matched to a control group (*n* = 59) and were recorded with 58 EEG electrodes during a visual counting oddball task. Three ERP components were extracted (i.e., P200, N200, and P300), and generating sources were modelized with Standardized Low-Resolution Electromagnetic Tomography. **Results**: We showed no group differences for the P200 and N200 when controlling for anxiety and depressive symptoms, suggesting that the early cognitive processes reflected by these components are relatively intact in these populations. Our results also showed a decrease in the later anterior P300 oddball effect for the TS and OCD groups, whereas an intact oddball effect was observed for the BFRB group. Source localization analyses with sLORETA revealed activations in the lingual and middle occipital gyrus for the OCD group, distinguishing it from the other two clinical groups and the controls. **Conclusions**: It seems that both TS and OCD groups share deficits in anterior P300 activation but reflect distinct brain-generating source activations.

## 1. Introduction

Gilles de la Tourette Syndrome (TS) is a neurodevelopmental disorder characterized by multiple chronic motor tics and at least one phonic tic with onset before 18 years old. The prevalence in the general population varies between 0.77% and 1.1% [1] and could significantly impact the quality of life [2]. Chronic tics are repetitive, non-stereotyped movements or sounds that can vary in complexity, frequency, localization, and severity throughout one’s life [3]. These typical symptoms tend to appear around the age of 4 to 6, with a peak of severity at 10 to 12 years [4]. While most patients with TS show a sharp decrease in tic symptoms after puberty [4], tics can sometimes persist in adulthood, with greater severity and a poorer response to pharmacology [5].

Concerning the etiology of TS, it seems that there is an agreement pointing toward broad structural and functional anomalies of the cortico-striato-thalamo-cortical circuit (CSTC), which form a loop including the basal ganglia, the thalamus, and the frontal cortex [6]. Other studies also point out anatomical and functional alterations of structures outside that circuit, particularly the ones forming the limbic system, such as the insula and the amygdala [7,8,9]. In addition, the supplementary motor area (SMA) seems to be affected in adult populations with TS [10], although this appears to be non-specific to TS [11]. It is well established that about 85% of individuals with TS present comorbidity with at least another disorder [12], pressing for more accuracy in group characterization and the specific contribution of some comorbidities in neurocognitive functioning.

One of the most common syndromes overlapping with TS in the adult population is obsessive compulsive disorder (OCD), which is characterized by obsessions and compulsions aiming to neutralize intrusive thoughts [13]. The most common obsessive thoughts are the fear of contamination, persistent doubts, aggressive, sexual, or religious thoughts, security and danger concerns, superstitions, and obsessions over symmetry [14,15]. The compulsions are behaviors or mental acts performed to prevent or reduce the distress associated with the obsessions, which could translate into over-verification, counting, repetition, and cleaning [14]. Neurobiological etiology is no exception to the similarities between OCD and TS because it appears that the CSTC loop is also involved in the pathology. Anatomical and functional anomalies have been observed in frontal regions like the orbitofrontal and prefrontal cortex [16,17,18,19].

Another syndrome that shares similarities with TS and OCD is Body-Focused Repetitive Behaviors (BFRB), also called habit disorders. BFRBs consist of specific behavior toward the body that occurs several times daily and can cause physical harm. The most common behaviors are onychophagia (nail-biting), trichotillomania (hair-pulling), and excoriation (skin-picking). Several attempts are often made to stop them [20], and these behaviors are generally voluntary, but people may experience a sense of loss of control over them [21]. Regarding the phenomenology of BFRB, it appears that a feeling of urge is often experienced before the execution of the habit, similar to that experienced by people addicted to certain substances, which suggests that both of these behaviors could be associated with the reward system [22]. Elaboration of the neurobiological BFRB etiology with neuroimaging studies on skin-picking and trichotillomania (hair-pulling) showed altered striatum volume and cortical thickness of the inferior frontal and orbitofrontal gyrus and rostral frontal gyri [23]. In addition, a smaller cortical thickness of the supramarginal gyrus and inferior parietal and temporal gyrus regions were associated with more symptom severity [24]. With that in mind, it is proposed that the smaller gray matter volumes found in the orbitofrontal cortex could play a reinforcement role as an insular input, which in turn is associated with interoceptive processes in the cerebellum, involved in cognitive-affective and motor functions that could explain symptom expression in BFRB subgroups [25,26].

These results underlined similarities and differences in the symptomology between BFRBs and other obsessive compulsive and related disorders. However, it is unclear whether we can relate these structures to the cognition and the information processing stream as assessed by functional brain imaging. If the structures delineated by the neuroimaging studies are differentially affected in TS, OCD, and BFRB, it is thus reasonable to infer that they also impact neurocognitive processing. To study these neurocognitive processes comprehensively, we need a measure sensitive to the information processing stream with a fast temporal resolution to infer which process is affected and what pertains to which group. The so-called Event-Related Potentials (ERPs) technique based on electroencephalography (EEG) constitutes an excellent candidate for addressing that question. ERPs are primarily used to monitor brain activity time-locked with cognitive processes. The ERP components are identified by their valence (N for negative and P for positive) and time interval in milliseconds (e.g., P200 for positivity at approximately 200 ms). We propose studying three ERP components, the P200, the N200, and the P300, elicited during a visual counting oddball task. Earlier studies revealed that the P200 amplitude, an index of early perceptual processing, seems relatively intact in the TS population [26,27,28] and BFRB [27]. The next component, the so-called N200 component, is believed to be an index of cognitive control more prominent in a motor task [28], which includes mismatch detection, response strategy regulation, and response inhibition [29]. Most of the studies reported an absence of N200 difference between TS and the control group [27,30,31], but other studies found a smaller [32] or a larger [33] N200 oddball effect in the TS group. A third component of interest, the P300, is implied to be involved in stimuli evaluation and categorization, particularly in working memory [28,34] and context updating [35]. It appears that there are some inconsistencies with the P300, where many studies found no difference in the P300 amplitude in TS [30,32,36,37] or a tendency toward a reduction [38] or a smaller P300 during an oddball [27,33] or a dual-task paradigm [39]. Some discrepancies could be ascribed to task modalities or specific instructions to the participant, eliciting a P300 related to stimulus detection, categorization, and cognitive flexibility. However, other discrepancies could also be related to possible comorbidities within the TS group, such as OCD or other concomitant disorders. Earlier studies already showed specific ERP anomalies in OCD.

Applied in various experimental paradigms in OCD groups, many researchers also found equivocal results for the P200 component throughout the years, where smaller [40], larger [41], or no amplitude differences [42,43] have been observed. Concerning the N200, a more consistent pattern was observed with a larger N200 amplitude [42,44] negatively correlated with OCD symptomatology [45]. Conflicting results have been obtained for the P300 component, where some studies reported a larger [46,47] or a reduced P300 amplitude [43,48,49,50] in adults with OCD.

In contrast to TS and OCD mentioned above, minimal ERP literature exists on BFRB. Thus, few studies have investigated these three components during an oddball paradigm. Morand-Beaulieu et al. [27] found no group difference in P200 amplitude when comparing the BFRB group to TS and a paired control group. This same study showed increased N200 amplitude in response to the frequent condition, decreasing the oddball effect. Finally, they observed a decrease in the P300 amplitude for the BFRB group in the non-motor variant of the task, while no intergroup difference was observed for the motor variant [27]. In contrast, one study also reported a decrease in P300 for the motor variant of an oddball task [38].

### 1.1. What Are the Main Contrasts and Commonalities across TS, OCD and BFRB?

We can discern several differences and similarities between these three disorders. From a psychophysiological standpoint, it appears that a feeling of urge is often experienced before the execution of the habit in BFRB or tics in TS. For instance, specific situations can cause symptom intensification where there is a reward system, specifically of negative reinforcement, involved in maintaining the behaviors; a feeling of relief is usually felt after their execution, and on an anatomical and functional level, it seems that the CSTC loop is involved in the symptomatology of these disorders. Furthermore, it has been reported that executing BFRB habits leads to pleasure and immediate gratification [51,52] and a feeling of release once the inner tension is gone [52,53], which could share similarities with the tension release after tic generation. Because they share many phenomenological similarities, it has been proposed that TS and BFRB form a close cluster [53]. These disorders are characterized by difficulties controlling actions, especially when inhibiting them [54]. From a neurobiological standpoint, interesting findings suggested commonalities between BFRB and TS with parts of the CSTC circuits involved. Specifically, with trichotillomania, a reduced cortical volume was detected initially in the inferior frontal gyrus, whereas a larger volume was observed in the cuneus [55], in the putamen [56], and in the cerebellum [57]. BFRB seems to have a more remarkable resemblance to tics disorders concerning the style of planning action, as observed in a study using the STOP questionnaire, which might situate BFRB between tics disorders and OCD on a symptomatic continuum [54]. Finally, a review examined the resemblances between trichotillomania, tic disorders, and OCD [58]. As the authors of this review stated, the response to treatment in trichotillomania, for instance, with antipsychotics and psychotherapies, is also more similar to the one in TS [59,60,61] than in OCD. Moreover, it seems that this observation extends to other kinds of BFRB, as shown in a study comparing the effect of cognitive behavioral therapy on a TS group and a BFRB group, the latter being composed of patients with trichotillomania but also with onychophagia, skin-picking, and bruxism [27]. Their results showed similar efficacity of this treatment on the symptoms of the two clinical groups.

With the OCD-BRFB comparison, it is believed that people with BFRB have difficulties with emotional regulation, and habits help them deal with their perceived negative state, which may contribute to maintaining behaviors through a reinforcement loop [62]. Because it is also largely believed that a reinforcement system might be involved in the phenomenology of OCD [63], this could represent a common expression between those two disorders. Indeed, relief is often reported after executing the compulsion, which might reinforce the person in maintaining those behaviors whenever they experience the negative feelings associated with the obsessions [63], which could also be similar to tic expression in TS. Moreover, other similarities seem to exist between TS and OCD regarding phenomenology. Indeed, while the sensation preceding tics in TS, called premonitory urge, is generally described by patients as physical tension or discomfort, it can sometimes take the appearance of a more cognitive feeling, namely a “not-just-right experience” [64]. It refers to a feeling that something is not just as it should be [65]. Mainly present in OCD, along with the feeling of incompleteness until a compulsion is judged correctly carried out, in TS, it could take the form of repeating a tic or a specific sequence of tics a certain number of times until that feeling is achieved [66]. This repetitive sequencing could mostly take place in a specific phenotype of what is believed to be “Tourettic OCD”, a hybrid condition between OCD and TS. That disorder could be distinguished from the two others as it presents a better response to a combined treatment for both tics and obsessive compulsive symptoms and presents very intertwined TS and OCD manifestations that could not be explained just by comorbidity [67,68]. This specific condition pinpoints the profound overlap between these two disorders and therefore accentuates the plausible common ground in which they occur, reinforcing the idea of a TS-OCD spectrum. The “just-right” feeling might also be present in BFRB, particularly trichotillomania. Indeed, an urge to pull hair has been associated with perfectionism, a concept that could be close to the just-right experience [58].

On the brain similarities between OCD and TS, a recent review revealed that the SMA seems to represent an essential target for repetitive transcranial magnetic stimulation in patients with OCD and TS [69]. This shared target could reveal common cerebral structures affected in these two groups. However, there are differences between these groups: the putamen and the sensorimotor cortex showed more connectivity in TS and were associated with more tic severity, whereas OCD severity was associated with decreased connectivity between the SMA and thalamus and between the caudate and the precuneus [11].

As highlighted by Lamothe et al., all these similarities between OCD, BFRB, and TS clearly show the importance of studying these disorders together, as they may be part of the same spectrum. This strategy would allow us to offer a better understanding of each disorder individually and help improve the efficacity of existing treatments or develop new ones for more refractory cases [58].

### 1.2. Objectives and Hypotheses

Our first aim is to characterize ‘when’ in the information processing stream group differences are the most prominent. This temporal characterization could allow us to understand these groups’ ERP differences and similarities. To address that question, we propose three ERP components in response to a visual counting oddball task. They are the reflection of three processing levels, namely the P200 (early evaluation of task-related stimuli), the N200 (attention orientation), and the P300 (working memory), to identify specific cognitive function alterations in the clinical groups. These components arise primarily between 200 and 400 ms post-stimulus. By that procedure, we propose to situate these groups in a TS-BFRB-OCD continuum while considering comorbid symptoms of anxiety and depression as a covariate. The second aim is to identify ‘where’ in the brain the group discrepancies could be. To address that question, we propose to model the source of the P300 oddball effect across each group with the Standardized Low-Resolution Electromagnetic Tomography (sLORETA) method. The TS, OCD, and BFRB were often compared two by two but never together.

Based on past literature, we hypothesize that (1) TS and BFRB groups will show reduced P300 amplitude [27,33,38] and that the P300 generators will be located in the temporoparietal areas, such as the supramarginal gyrus [38], whereas the OCD group will show a more significant P300 oddball effect with generators localized in the orbitofrontal cortex and posterior parietal regions [70]; (2) we do not expect differences across the three clinical groups and the control group for the P200 component [27,30,42,43]; (3) we also hypothesize that a more significant N200 oddball effect will be observed for the OCD group [42,44,45], a greater N200 amplitude for the frequent condition in the BFRB group [27], and a similar pattern between the TS group and the control group [27,30,31].

## 2. Materials and Methods

### 2.1. Participants

Twenty-four participants were included in the TS group, eighteen participants in the OCD group, and sixteen participants in the BFRB group. These three groups (*n* = 58) were matched to fifty-nine control participants on age and nonverbal intelligence. Participants were excluded from this study in case of other psychiatric disorders, such as schizophrenia, bipolar disorder, somatoform disorders, dissociative disorders, and substance-related disorders, as they could have an impact on the results and are not considered part of the TS spectrum. The presence of personality disorders was screened with the French version of the personality diagnostic questionnaire 4th Ed [71,72].

In addition, a neurological assessment was performed by P.J.B., a neurologist specializing in movement disorders, who was responsible for the differential diagnosis and therefore had to exclude any participant with other medical conditions, particularly neurological diagnoses such as Parkinson’s, hemifacial spasms, myoclonus, neuroacanthocytosis, Meige syndrome, cerebral sclerosis, Huntington’s, and Wilson’s disease. Another exclusion criterion included unstable medications (i.e., change in medication over the last three months). Participants were also excluded from our sample if they presented altered EEG signals or if some technical difficulties emerged during data acquisition, which explains the disproportionate group size. The general inclusion criteria for all participants were that participants must have normal or corrected to normal visual acuity (Snellen notation system) and be 18 years or older.

Data acquisition was conducted at the Laboratoire de psychophysiologie cognitive & sociale of the Centre de recherche de l’Institut universitaire en Santé mentale de Montréal (CR-IUSMM) by a qualified technician. The following tools were administered by clinician psychologists, except for the Beck Anxiety and Depression Inventories, which are self-report questionnaires. All data presented in the current study were acquired before the beginning of specialized therapy to manage symptoms in the clinical groups.

### 2.2. Instruments and Clinical Assessment

To assess tic severity, the TS group undertook the Yale Global Tic Severity Scale (YGTSS) [73]. This is a 15–20 min semi-structured interview to collect information on tics’ specificity and anatomical distribution over one week. The questionnaire comprises five distinct dimensions (number, frequency, intensity, complexity, and interference). A 6-anchors ordinal scale was developed for each of the five dimensions, with each anchor corresponding to a relevant example or descriptive statement. The YGTSS also includes an assessment of the impact of the tic disorder over the past week. It encompasses self-perception and self-esteem, relationships with close family members, social relationships, and academic and occupational performance skills. This assessment is measured using a six-point ordinal scale. The YGTSS global score has a maximum of 100 and is the sum of the two subscales evaluating the severity of tics (0–50) and the daily impairment (0–50). In general, this measurement tool has psychometric properties considered excellent in terms of internal consistency coefficients (α = 0.91) [74], test–retest reliability (ICC = 0.89) [74], inter-rater agreement (between ICC = 0.62 and ICC = 0.85) [73], and convergent validity with the Tourette Syndrome Global Scale (TSGS) between r = 0.86 and r = 0.90 [75].

An adapted version of this scale [21] was used to measure the severity of BFRB, where the word “tic” is replaced by the word “habit”. The psychometric properties of this tool are the following: internal consistency coefficient of α = 0.86 and test–retest reliability (ICC = 0.70).

The Yale–Brown Obsessive Compulsive Scale (YBOCS) [76] was administered to the OCD group to assess the obsessive compulsive symptom severity. The 10-item scale comprises four anchors, ranging from 0 (i.e., no symptoms) to 4 (i.e., extreme symptoms), with a total of 40 points. Two sub-scores were calculated: items 1 to 5 correspond to the severity of obsessive symptoms, and items 6 to 10 to the severity of compulsive symptoms. The scale is based on five dimensions: time, interference, distress, resistance, and control. The validity and reliability of this instrument were confirmed by other studies (internal consistency = 0.91–0.94, r = 0.90) [76,77,78].

All participants completed the Beck Anxiety Inventory (BAI) [79] to evaluate their level of anxiety symptoms. The BAI is a 21-item self-report scale used to measure the level of anxiety, where each item describes a common anxiety symptom. The participant is asked to report, on a scale ranging from 0 (i.e., no symptoms) to 4 (i.e., severe), the extent to which they have experienced the symptom over the past week. The total score can range from 0 to 63 points. BAI scores are classified as sub-clinical anxiety (0 to 7), mild anxiety (8 to 15), moderate anxiety (16 to 25), and severe anxiety (26 to 63). This measurement tool has psychometric properties deemed acceptable, with an internal consistency coefficient of α = 0.84 and test–retest reliability of around r = 0.63 [80].

Participants also completed the Beck Depression Inventory (BDI) [81,82], a 21-item self-report scale, to measure their level of depression, each item describing a common depressive symptom. Each item comprises 3 points ranging from 0 to 3 to indicate the severity of the symptom. BDI-II scores are classified as minimal depression (0 to 9), mild depression (10 to 18), moderate depression (19 to 29), and severe depression (30 to 63). Psychometric properties of the French version of this instrument are considered acceptable, with an internal coherence coefficient of α = 0.92–0.93 and a test–retest reliability coefficient of α = 0.93.

Raven’s progressive matrices [83] composed a non-verbal intelligence test in which the participant must identify the missing element in a matrix according to a certain logic. The short version was administered to all participants. The “Split-half” reliability obtained reports results around r > 0.90.

The Edinburgh Handedness Inventory [84] is a ten-item questionnaire that quantitatively measures laterality. The participant is asked with which hand they carry out various activities of daily life, such as writing, drawing, cutting with scissors, brushing teeth, etc., which ultimately results in a laterality quotient (i.e., a score), indicating left-handedness, right-handedness, or ambidexterity.

### 2.3. Procedures

A counting oddball task was used for all groups while their electrophysiological activity was recorded with an EEG. This experimental task comprises two conditions: the frequent, which composes 80% of the trials (*n* = 160), and the rare, which occurs in the 20% remaining (*n* = 40). The frequent stimuli (letter « O ») and the rare stimuli (letter « X ») are randomly presented during 100 ms at the center of a 17-inch Viewsonic SVGA computer screen. Each stimulus is separated by 1700 to 2200 ms interstimulus randomized intervals. The order of presentation of the two types of stimuli is counterbalanced. Participants were seated with their heads at about 90 cm from the monitor at a 5-degree horizontal angle. They must look at the fixation cross, count the rare stimuli, and report that exact number at the end of the task. No motor response is required. The entire duration of the task is eight minutes. Correct task performance only depended on one criterion: whether the participant counted the right amount of rare stimuli (i.e., 40).

### 2.4. Electrophysiological Recordings

EEG signals were recorded using a digital amplifier (Sensorium Inc., Charlotte, VT, USA) via 58 silver/silver chloride electrodes arranged on nylon/lycra cap (Electrode Arrays, El Paso, TX, USA following standard EEG procedures according to the extended 10–20 system [85] with the common reference electrode placed on the nose. EEGs were recorded continuously at a frequency of 500 Hz and then filtered by a 0.01 Hz high-pass filter, a 100 Hz low-pass filter, and a 60 Hz Notch filter. Signal resistance was kept below five kΩ using a conductive gel (JNetDirect Biosciences, Herndon, VA, USA). A bipolar electro-oculogram (EOG) was recorded to clear EEG from eye artifacts. EOGs were placed at the outer canthus of each eye (horizontal EOG) and infra-supra-orbital to the left eye (vertical EOG). All electrodes were referenced to the nose. The stimuli were monitored by Presentation (Neurobehavioral Systems, Albany, CA, USA http://www.neurobs.com/ (accessed on 17 June 2018), while signal acquisition was controlled by IWave version 7 from InstEP Systems, Montréal, QC, Canada) running on two PCs.

### 2.5. EEG Signal Extraction

Ocular artifacts were corrected offline using the Gratton algorithm [86]. Raw EEG signals were averaged offline, time-locked to the stimulus onset, from 100 ms before to 1500 ms after stimulus onset. Clippings due to amplifier saturation and remaining epochs exceeding ±100 uV were removed during the averaging procedure. Epochs containing less than 20 trials for each category were excluded. Both the P200 and N200 amplitudes are the positive (P200) and negative (N200) valence ERP components located in the 150–300 ms time window after stimulus onset. For the P300 component, it is the maximum amplitude of positive valence, occurring between 300 and 550 ms post-stimulus.

Fifty electrodes were used for the mixed ANOVAs (see the section on statistics for details). Among these 50 electrodes, we find seven groupings (regions): the anterofrontal consisting of electrodes AF1, AF2, AF3, AF4, AF7, AF8, and AFZ; the frontal consisting of electrodes F1, F2, F3, F4, F5, F6, and FZ; the frontocentral consisting of electrodes FC1, FC2, FC3, FC4, FC5, FC6, and FCZ; the central consisting of electrodes C1, C2, C3, C4, C5, C6, and CZ; the centroparietal consisting of electrodes CP1, CP2, CP3, CP4, CP5, CP6, and CPZ; the parietal consisting of electrodes P1, P2, P3, P4, P5, P6, and PZ; and finally the parieto-occipital region consisting of electrodes PO1, PO2, PO7, PO8, TP7, TP8, POZ, and OZ.

For analyses performed via the sLORETA method (Standardized Low-Resolution Electromagnetic Tomography; University Hospital for Psychiatry, Zürich, Switzerland, https://www.uzh.ch/keyinst/loreta.htm) (accessed on 1 December 2023), we employed all 58 electrodes, which include AF1, AF2, AF3, AF4, AF7, AF8, AFZ, C1, C2, C3, C4, C5, C6, CP1, CP2, CP5, CP6, CPZ, CZ, F1, F2, F3, F4, F5, F6, F7, F8, FZ, FC1, FC2, FC3, FC4, FC5, FC6, FCZ, FT7, FT8, O1, O2, OZ, P1, P2, P3, P4, P5, P6, PO1, PO2, PO7, PO8, POZ, PZ, T3, T4, T5, T6, TP7, and TP8. They are arranged according to the extended international 10/20 system [87] used to maximize the localization of the source densities of the P300 component.

### 2.6. Statistical Analysis

Statistical analyses were performed with IBM SPSS Statistics version 26 software (IBM Corp., New York, NY, USA). First, one-way analyses of variance (One-Way ANOVA) were performed on age, gender, intelligence (Raven’s Progressive Matrices), depression (BDI), anxiety (BAI), and laterality (Edinburgh Handedness Inventory). These analyses ensure that the four groups are comparable. Games–Howell tests were used to compare groups with heterogeneous variances. In addition, independent samples *t*-tests were used to compare the YGTSS scores of the TS and BFRB groups.

Where we found group differences in anxiety or depression, we plan to add the anxiety (BAI) and depression (BDI) covariations to the analyses in order to test whether these symptom dimensions impact the results in the mixed ANOVAs.

In order to compare the N200, P200, and P300 components between each group, mixed ANOVAs were performed, where a between-group factor was used with TS, OCD, BFRB, and control (four levels). The following within-group factors were also used with conditions (two levels: frequent and rare), hemispheres (two levels: right and left), regions (seven levels: anterior frontal (AF), frontal (F), frontal central (FC), central (C), central parietal CP), and parietal (P)). Similar analyses were performed on the eight midline electrodes (midline Z). This analysis was performed with the same inter-group factor and with the following intra-group factors: conditions (with two levels: frequent and rare) and electrodes (with eight levels: AFZ, FZ, FCZ, CZ, CPZ, PZ, POZ, and OZ). BDI and BAI scores were used as covariables to observe if higher levels of anxiety and depressive symptoms in the clinical populations would influence the observed amplitude differences. The significance threshold was defined at 5% for all analyses.

### 2.7. Source Localization

Following the statistical analyses mentioned above, analyses with the sLORETA method were carried out to perform source localization analyses. These were performed on the time window corresponding to the P300 component (300 to 550 ms post-stimulus). As specified by [88], this method requires several electrodes covering a maximum of brain regions, so we increased the number of electrodes to 58. sLORETA uses a distributed source localization algorithm to solve the inverse problem of brain electrical activity [89], regardless of the number of neuronal generators [89,90]. The sLORETA algorithm calculates the current density values (unit: amperes per square meter; A/m^2^) of 6239 gray matter (GM) volume voxels belonging to a brain compartment with a spatial resolution of 5 mm × 5 mm × 5 mm each. The three-dimensional brain compartment includes only cortical gray matter and the hippocampus and contains no deep brain structures such as the thalamus or cerebellum. Anatomical regions are labeled according to (1) the MNI-152 probabilistic model made digitally available by the Brain Imaging Center of the Montreal Neurological Institute [91] and (2) Talairach [92]—a digitized version of the Coplanar Stereotactic Atlas of the Human Brain introduced by Talairach and Tournoux [93].

The first step is determining the time window (i.e., when) corresponding to the component in a given condition by performing *t*-tests contrasting each group’s frequent and rare conditions consistent with an oddball paradigm. Thus, the following parameters were used first to identify the time course of the oddball effect within each group: Data type: ERP|Reference: None|Number of electrodes: 58|Number of Timeframes: 500|Data scaling/Normalization: Subject-wise|Test/Analysis: Paired group, test A = B|Baseline correction: None|Test details: All Tests for all Time Frames|Parameters for statistical analysis: t-statistic|Perform randomization SnPM, and compute bullet proof corrected critical thresholds and *p* values|number of randomization: 5000. A narrow time interval was thus identified for each group, which corresponds to the time window of the optimized and significant *t*-tests between the rare and frequent conditions. The width of these time windows was determined based on similar t-values.

The second step is to estimate the location (i.e., where) of the generators for the oddball effects of each group separately. To display the differences between the two experimental conditions, for the P300 time window (e.g., “rare” > “frequent” for the oddball paradigm), nonparametric statistical mapping (SnPM), as introduced by Nichols and Holmes [94] was used to calculate the mean distribution of intracerebral current density at time intervals, showing significant differences based on nonparametric matched samples on the mean log F-ratio (log F-ratio), including 5000 randomized permutations on the three-dimensional sLORETA images. Statistical significance was assessed by defining corrected critical thresholds (critical t) for multiple comparisons (*p* < 0.01 and (*p* < 0.01 and *p* < 0.05, respectively) for all voxels tested. Null hypotheses were equivalent to those with no differences between experimental conditions. Current density values at each voxel were calculated as a linear, weighted sum of the electrical potentials. The activation of a given voxel was based on the smoothness assumption, where neighboring voxels show highly synchronous activity [95]. Thus, electrophysiological studies show that neighbouring neuronal populations exhibit highly correlated electrical activity [95,96]. Activated voxels exceeding critical t-values were considered regions of significant cortical activation [97]. Statistical analysis then resulted in a corresponding three-dimensional average intracerebral current density distribution and cortical regions were classified according to Brodmann areas [98] and their corresponding normalized coordinates (Talairach and MNI, respectively).

## 3. Results

### 3.1. Sociodemographic and Clinical Data

No significant difference has been found for age, laterality, and intelligence (Table 1), but significant differences have been found for sex (*p* < 0.01) within the BFRB group and the three other groups, with a higher proportion for females. This sex effect in BFRB is not surprising because more women are affected by this condition. Significant differences have also been found for anxiety (BAI) and depression (BDI) scores, distinguishing the control group from the three others. *t*-tests for independent samples also showed a significant difference in YGTSS scores between TS and BFRB groups (T = 2.52 (*p* < 0.05)).

### 3.2. Event-Related Potentials

#### 3.2.1. P200 Amplitude

Maximum P200 amplitude was observed at a 224 ms post-stimulus mean latency. The mixed ANOVA showed significant main effects for condition (F [1,113] = 39.94, *p* < 0.001, power = 1, η2partial = 0.261) and region (F [6,108] = 14.49, *p* < 0.001, power = 1, η2partial = 0.45), a condition by region interaction (F [6,108] = 39.24, *p* < 0.001, power = 1, η2partial = 0.69) and a Condition by Region by Hemisphere interaction (F [6,108] = 2.48, *p* < 0.05, power = 0.81, η2partial = 0.12) which indicates that the P200 oddball effect is more prominent in the central region of the right hemisphere.

Group differences were also found with a group by condition interaction (F [3,113] = 3.13, *p* < 0.05, power = 0.72, η2partial = 0.077). Because intergroup interactions were obtained, subsidiary ANOVA decomposed by groups showed main effects for the condition in the TS (F [1,23] = 19.26, *p* < 0.001, power = 0.987, η2partial = 0.456), BFRB (F [1,15] = 14.87, *p* = 0.002, power = 0.949, η2partial = 0.498), and OCD (F [1,17] = 26.49, *p* < 0.001, η2partial = 0.609). The P200 condition effect failed to reach significance in the control group (F [1,58] = 2.59, *p* = 0.113, power = 0.353, η2partial = 0.043). Thus, the condition effect was significant in all three clinical groups, where a larger P200 was in response to the rare condition. However, the Group by Condition was no longer significant when adding the BDI or the BAI (*p* = 0.17) score as a covariable.

A main effect for Hemisphere (F [1,113] =16.96, *p* < 0.001, power = 98, η2partial = 0.13), a double interaction Hemisphere by Group interaction (F [3,113] = 3.27, *p* < 0.05, power = 0.74, η2partial = 0.08). Subsidiary ANOVAs revealed a more significant right Hemisphere effect for OCD (F [1,17] = 14.55, *p* = 0.001, power = 0.949, η2partial = 0.461), Controls (F [1,58] = 14.06, *p* < 0.001, power = 0.958, η2partial = 0.195), and BFRB (F [1,15] = 5.42, *p* = 0.034, power = 0.586, η2partial = 0.265), respectively. This hemisphere effect shows hemispheric lateralization in the right hemisphere for all groups except for TS. This interaction remained significant after covarying with depression (Hemisphere by Group [F (3,109) = 3.16, *p* < 0.05, power = 0.719, η2partial = 0.08]) or anxiety (Hemisphere by Group (F [3,109] = 2.91, *p* < 0.05, power = 0.679, η2partial = 0.074).

The mixed ANOVA performed on the central electrodes (AFZ, FZ, FCZ, CZ, CPZ, PZ, POZ, and OZ) revealed significant main effects for Condition (F [1,113] = 40.43, *p* < 0.001, power = 1, η2partial = 0.264) and Electrodes (F [7,107] = 19.56, *p* < 0.001, power = 1, η2partial = 0.561). In addition, a Condition by Electrodes was observed (F [7,107] = 29.99, *p* < 0.001, power = 1, η2partial = 0.662), suggesting that conditions differed for some electrodes in all groups. However, no significant interaction with the group factor was observed, suggesting no inter-group difference for the mid-electrodes.

#### 3.2.2. N200 Amplitude

The maximum amplitude of the N200 component was observed at a mean latency of 224 ms post-stimulus. Mixed ANOVA revealed significant main effects for Region (F [6,108] = 15.98, *p* < 0.001, η2partial = 0.47) and Hemisphere (F [1,113] = 13.03, *p* < 0.001, η2partial = 0.103). A Condition by Region (F [6,108] = 18.39, *p* < 0.001, power = 1, η2partial = 0.505) interaction was present, indicating a more prominent N200 oddball effect over the anterior frontal and frontal regions. In addition, a significant Condition by Hemisphere interaction (F [1,113] = 10.34, *p* < 0.005, power = 0.89, η2partial = 0.084) shows a hemispheric difference between the two conditions. Finally, a triple Condition by Region by Hemisphere interaction (F [6,108] = 6.49, *p* < 0.001, power = 0.999, η2partial = 0.265) was obtained, which showed a more significant N200 oddball effect over the right hemisphere (left or right hemisphere) at the fronto-central region.

A group effect was found with a significant Group by Condition interaction (F [3,113] = 3.25, *p* < 0.05, power = 0.73, η2partial = 0.079). The subsidiary ANOVAs were decomposed by group, and this revealed condition effects for OCD (F [1,17] = 7.17, *p* = 0.016, power = 0.713, η2partial = 0.297) and controls (F [1,58] = 5.69, *p* = 0.02, power = 0.65, η2partial = 0.089), where a larger amplitude N200 is observed for the rare condition. The N200 oddball effect was insignificant in the BFRB and TS groups. However, with the addition of the BDI (*p* = 0.087) or the BAI (*p* = 0.104) as a covariate, the Condition by Group interaction is no longer significant. In summary, scores on the BDI and BAI appear to impact the N200 amplitude.

The mixed ANOVA performed on the mid-electrode line showed significant main effects for Electrodes (F [7,107] = 12.78, *p* < 0.001, power = 1, η2partial = 0.455). A double interaction was significant in either Condition by Electrodes (F [6,107] = 16.54, *p* < 0.001, power = 1, η2partial = 0.52), suggesting a difference between the two conditions for some electrodes. No group difference was noted over the midline electrodes.

#### 3.2.3. P300 Amplitude

Maximum P300 amplitude has been observed at a 408 ms post-stimulus mean latency for all participants. Significant main effects for the mixed ANOVA were found for Condition (F [1,113] = 243.80, *p* < 0.001, power =1, η2partial = 0.68), Region (F [6,108] = 38.25, *p* < 0.001, power = 1, η2partial = 0.68), and Hemisphere (F [1,113] = 6.38, *p* < 0.05, power = 0.70, η2partial = 0.05). A significant Condition by Region interaction (F [6,108] = 87.04, *p* < 0.001, power = 1, η2partial = 0.83) and a Hemisphere by Region interaction (F [6,108] = 11.02, *p* < 0.001, power = 1, η2partial = 0.38) were found. A significant Condition by Region by Hemisphere interaction (F [6,108] = 21.42, *p* < 0.001, power = 1, η2partial = 0.54) was obtained.

**Figure 1:**
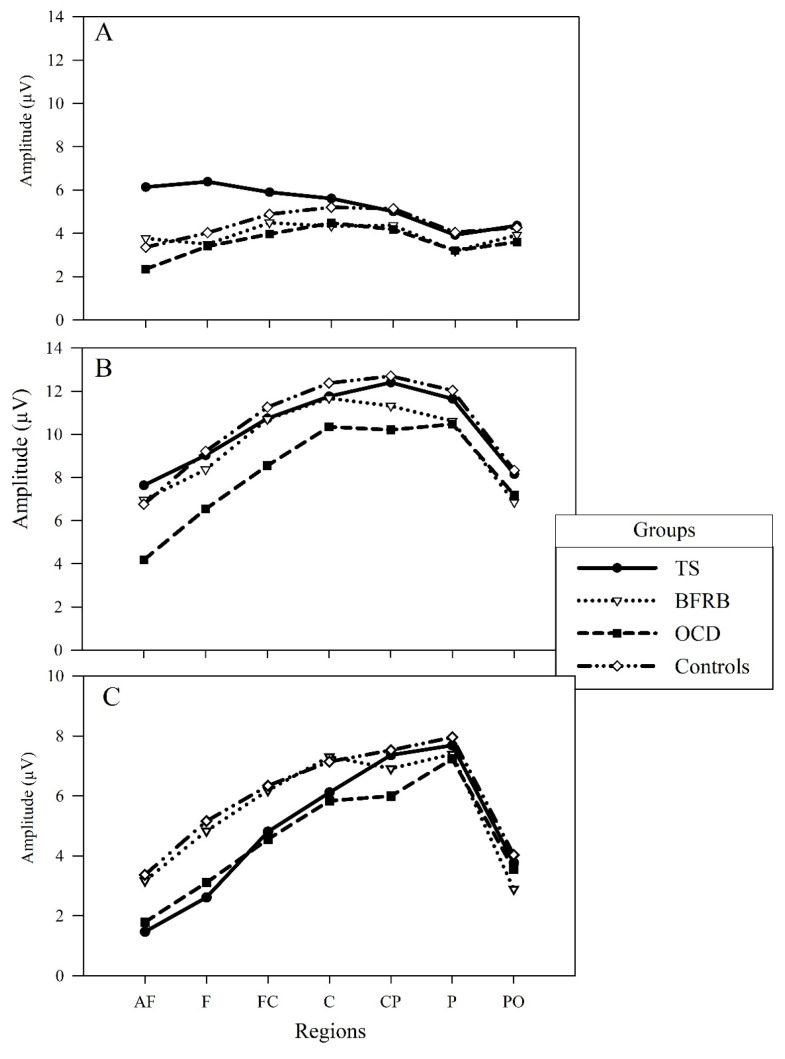
Condition by Region by Group interaction of the P300 component. Intergroup comparison of the amplitude of the frequent (**A**), the rare (**B**) conditions, and the oddball P300 effect (**C**), which represent the rare minus frequent subtraction. Amplitude in microvolts (μV) composes the *y*-axis, and regions compose the *x*-axis. The TS group shows a larger amplitude in frontal regions in the frequent condition, while the OCD group shows a more distributed amplitude decrease in the rare condition. These two different activation patterns result in a decreased oddball P300 effect for the TS and OCD groups compared to the control and BFRB groups. AF: antero-frontal; F: frontal; FC: fronto-central; C: central; CP: centroparietal; P: parietal; PO: parieto-occipital.

The group interaction was found with Group by Condition by Region (F [18,330] = 1.71, *p* < 0.05, power = 0.949, η2partial = 0.085) (Figure 1), which shows that P300 centroparietal oddball effect is differently distributed for specific groups. When covaried with the BAI score, the Group by Condition by Region interaction remained significant (F [18,318] = 1.74, *p* < 0.05, power = 0.953, η2partial = 0.09)), suggesting that anxiety symptoms did not affect P300 amplitude. However, when covariation with the BDI score, the Group by Condition by Region revealed only a tendency (*p* = 0.07), suggesting that depressive symptoms explain in part our intergroup effects.

Therefore, a subsidiary ANOVA by group was applied and showed a significant Condition by Region interaction for TS (F [6,18] = 60.60, *p* < 0.001, power = 1, η2partial = 0.953), OCD (F [6,12] = 65.93, *p* < 0.001, power = 1, η2partial = 0.971), control (F [6,49] = 24.43, *p* < 0.001, power = 1, η2partial = 0.749), and BFRB (F [6,10] = 13.03, *p* < 0.001, power = 1, η2partial = 0.887), respectively. However, inspection of the scalp distribution revealed a reduced oddball effect over anterior electrodes (AF, F, FC, and C regions) in the TS and OCD groups, whereas the BFRB and the controls have a more similar profile. We also found a significant Condition by Regions by Electrodes by Group interaction (F [36,312] = 1.78, *p* < 0.05, power = 0.998, η2partial = 0.171), which necessitates a deeper modelization of the generating sources.

The mixed ANOVA performed on the midline electrodes showed significant main effects for Condition (F [1,113] =309.80, *p* < 0.001, power = 1, η2partial = 0.733) and Electrodes (F [7,107] = 34.71, *p* < 0.001, power = 1, η2partial = 0.694). A Condition by Electrodes interaction (F [7,107] = 46.33, *p* < 0.001, power = 1, η2partial = 0.752), as well as an Electrodes by Group interaction (F [21,327] = 2.08, *p* = 0.004, power = 0.991, η2partial = 0.118), were obtained. A subsequent decomposition by the group revealed that the amplitude of the P300 differed for TS (F [7,17] = 8.24, *p* < 0.001, power = 0.999, η2partial = 0.772), BFRB (F [7,9] = 10.70, *p* < 0.001, power = 0.997, η2partial = 0.893), OCD (F [7,11] = 9.69, *p* < 0.001, power = 0.998, η2 partial = 0.860) and controls (F [7,52] = 31.58, *p* < 0.001, power = 1, η2partial = 0.810). Adding BDI or BAI scores as covariates did not affect the above Electrodes by Group interaction and remained significant.

#### 3.2.4. Source Localization of the P300 Oddball Effect

Statistical analyses with sLORETA on the rare > frequent contrast were performed separately within the P300 time window (i.e., between 300 and 550 ms after stimulus onset) for each group. Specifically, the mean latencies corresponding to the oddball effects of P300 for each group obtained in SPSS were used as benchmarks to identify the time window associated with this component in sLORETA.

**Figure 2:**
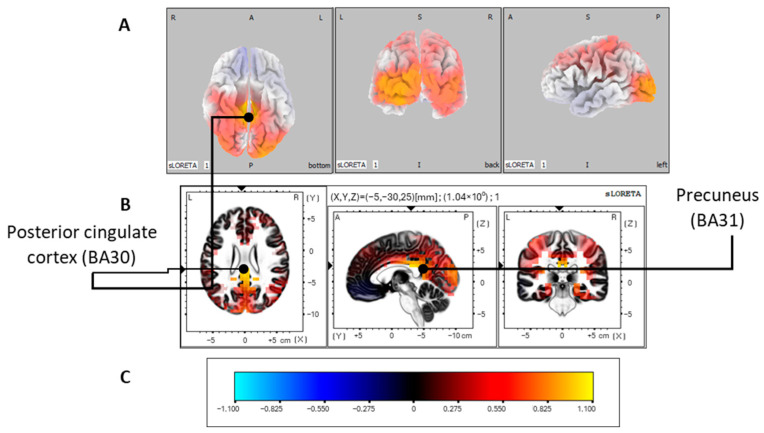
P300 oddball effect activations for the control group. Modelization obtained with sLORETA of the P300 oddball effect (contrast: rare > frequent) source localization of the control group in the 384 to 402 ms post-stimulus time window. Images were acquired after nonparametric statistical mapping (SnPM) and a Talairach stereostatic space coregistration based on a digitized version of the Coplanar Stereotactic Atlas of the Human Brain introduced by Talairach and Tournoux [93] and the MNI 152 probabilistic model made digitally available by the Brain Imaging Center of the Montreal Neurological Institute [91]. After corrections for multiple comparisons (*p* ≤ 0.05), activated voxels are shown in yellow. (**A**) Axial (ventral), coronal (caudal), and sagittal (left hemisphere) sections showing activations with the Colin27 three-dimensional model [99]. (**B**) The most significant activation was found in the posterior cingulate cortex (BA30). Significant activations were also found in the precuneus (BA31). (**C**) Color legend. L, left; R, right; A, anterior; P, posterior; MNI, Montreal Neurological Institute; X, Y, Z correspond to MNI coordinates; BA, Brodmann area.

Thus, the control group’s mean latency of 397 ms post-stimulus was observed. Consequently, we could identify a time window with sufficient global frequency power (GFP) ranging from 384 ms to 402 ms in sLORETA corresponding to the P300 window and a maximum surface activation difference in the middle occipital gyrus (BA19). Subsequently, source localization analyses on this time window revealed significant activation (*p* < 0.01) in the posterior cingulate cortex (BA30), which is localized in the limbic lobe. However, as shown in Table 2, other areas showed significant activation, such as the precuneus (BA31) in the parietal lobe (*p* < 0.05) (Figure 2).

**Figure 3:**
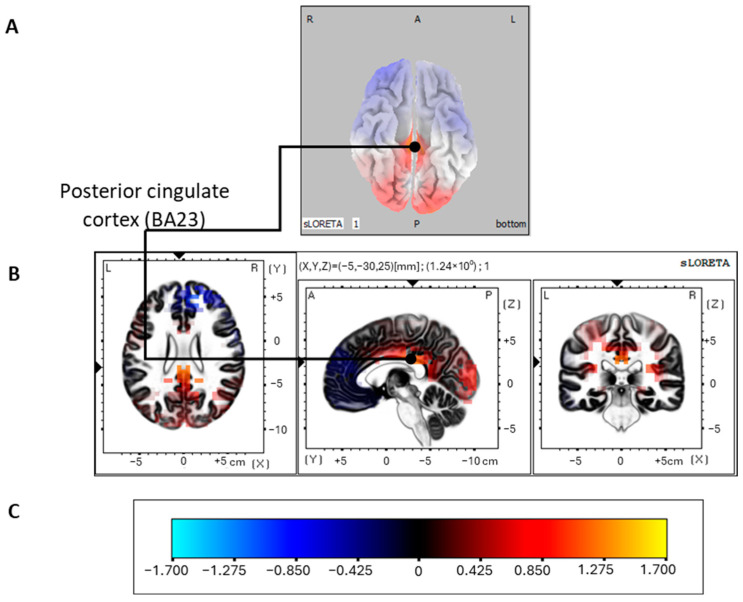
P300 oddball effect activations for the TS group. Modelizations obtained with sLORETA of the P300 oddball effect (contrast: rare > frequent) of the source localization of the TS group in the 376–396 ms post-stimulus time window. Images were acquired after nonparametric statistical mapping (SnPM) and a Talairach stereostatic space coregistration based on a digitized version of the Coplanar Stereotactic Atlas of the Human Brain introduced by Talairach and Tournoux [93] and the MNI-152 probabilistic model made digitally available by the Brain Imaging Center of the Montreal Neurological Institute [91]. No activation reached significance after corrections for multiple comparisons (*p* ≤ 0.05). (**A**) Axial (ventral) section showing activations with the three-dimensional Colin27 model [99]. (**B**) The most significant activation was found in the posterior cingulate cortex (BA23). (**C**) Color legend. L, left; R, right; A, anterior; P, posterior; MNI, Montreal Neurological Institute; X, Y, Z correspond to MNI coordinates; BA, Brodmann area.

For the TS group, we identified a temporal window ranging from 376 to 396 ms in sLORETA. Thus, a difference in maximum surface activation in the postcentral gyrus (BA07, parietal lobe) was observed. The generators of this oddball effect seem to be located in the posterior cingulate cortex (BA23) in the limbic lobe (Figure 3). However, these sources do not reach the threshold of significance (*p* between 0.05 and 0.10).

**Figure 4:**
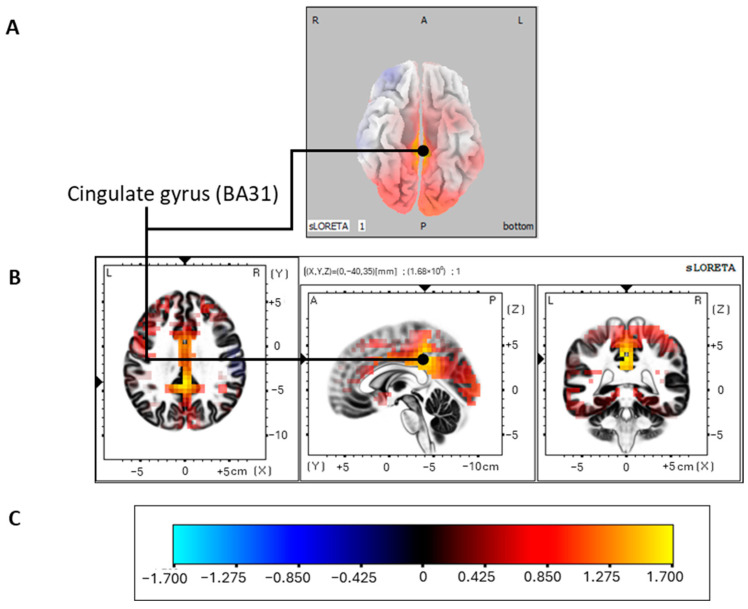
P300 oddball effect activations for the BFRB group. Modelizations obtained with sLORETA of the P300 oddball effect (contrast: rare > frequent) source localization of the BFRB group in the 408–428 ms post-stimulus time window. Images were acquired after nonparametric statistical mapping (SnPM) and a Talairach stereostatic space coregistration based on a digitized version of the Coplanar Stereotactic Atlas of the Human Brain introduced by Talairach and Tournoux [93] and the MNI-152 probabilistic model made digitally available by the Brain Imaging Center of the Montreal Neurological Institute [91]. After corrections for multiple comparisons (*p* ≤ 0.05), the yellow voxels activated. (**A**) Axial (ventral) section showing activations with the three-dimensional Colin27 model [99]. (**B**) The largest and only significant activation was found in the cingulate gyrus (BA31). (**C**) Color legend. L, left; R, right; A, anterior; P, posterior; MNI, Montreal Neurological Institute; X, Y, Z correspond to MNI coordinates; BA, Brodmann area.

For BFRB, because the mean latency of the P300 oddball effect observed in SPSS occurs at 419 ms post-stimulus, we selected in sLORETA a time window from 408 to 428 ms, where activations were identified in the middle occipital gyrus (BA19). Analyses revealed that the generators of this oddball effect for BFRB are found in the cingulate gyrus (BA31, limbic lobe; *p* = 0.05). This was the only area showing significant activation (Figure 4).

**Figure 5:**
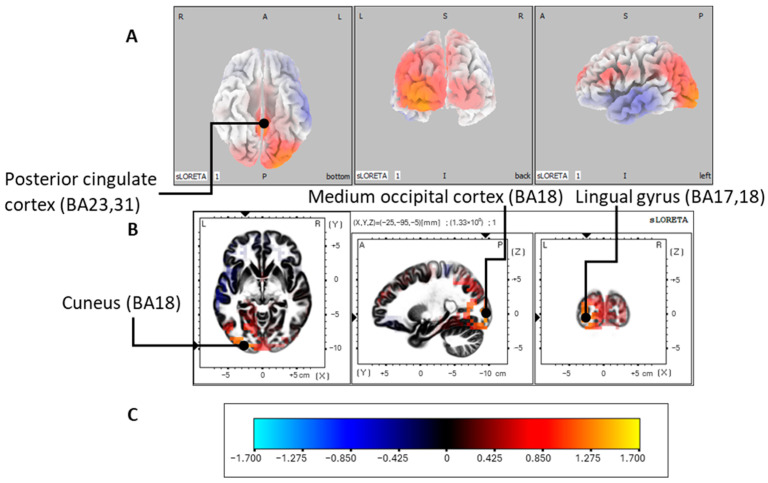
P300 oddball effect activations for the OCD group. Modelizations obtained with sLORETA of the P300 oddball effect (contrast: rare > frequent) source localization of the OCD group in the 402 to 418 ms post-stimulus time window. Images were acquired after nonparametric statistical mapping (SnPM) and a Talairach stereostatic space coregistration based on a digitized version of the Coplanar Stereotactic Atlas of the Human Brain introduced by Talairach and Tournoux [93] and the MNI-152 probabilistic model made digitally available by the Brain Imaging Center of the Montreal Neurological Institute [91]. After corrections for multiple comparisons (*p* ≤ 0.05), activated voxels are in orange. (**A**) Axial (ventral), coronal (caudal), and sagittal (left hemisphere) sections showing activations with the three-dimensional Colin27 model [99]. (**B**) The cuneus (BA18) was the most significant activation. Significant activations were also found in the lingual gyrus (BA17 and BA18), middle occipital gyrus (BA18), and posterior cingulate cortex (BA23,31). (**C**) Color legend. L, left; R, right; A, anterior; P, posterior; MNI, Montreal Neurological Institute; X, Y, Z correspond to MNI coordinates; BA, Brodmann area.

Finally, for OCD, the mean latency of the P300 oddball effect observed with the ANOVA in SPSS occurs at approximately 414 ms post-stimulus. With the sLORETA analysis, we therefore also focused our attention on a temporal window orbiting around this, i.e., between 402 and 418 ms post-stimulus, and observed a difference in maximal surface activation in the precuneus (BA19, parietal lobe). As shown in Figure 5, the generators of the P300 oddball effect for the OCD group are found in the cuneus (BA18, occipital lobe, *p* between 0.01 and 0.05), but also in other significant regions such as the lingual gyrus located in the occipital lobe as well, the middle occipital gyrus, the posterior cingulate cortex and the precuneus located in the parietal lobe (Table 3).

## 4. Discussion

Our first aim was to characterize ‘when’ in the information processing stream the group differences arise by looking at the N200, P200, and P300 components. Moreover, anxious and depressive symptoms were used as covariates to observe their potential impact on ERPs. Results for the P200 component revealed a significant oddball effect only in clinical groups. An interesting finding is that the presence of depression or anxiety attenuates that P200 oddball effect. Results for the N200 amplitude revealed that the oddball effect was significant for the control and OCD groups, whereas it was non-significant for the TS and BFRB groups. However, these group discrepancies were attenuated and non-significant again by the introduction of the covariance of anxiety or depression symptoms. Finally, the P300 is differently distributed across groups. More precisely, the P300 oddball effect is notably reduced over the anterior regions for the TS and OCD groups, whereas the control and BFRB groups were not different. This pattern remains significant after covarying for anxiety.

The P200 is typically associated with early stimuli evaluation and their task-related adequacy [100,101,102,103,104]. We first hypothesized that no group difference would be observed for the P200 oddball effect, which was confirmed by our results. When we controlled for anxiety and depression symptoms, we failed to detect a P200 oddball effect, which was true across all groups. This result could be explained by the characteristics that differentiate the target stimulus in our paradigm. Indeed, their treatment may involve cognitive processes beyond early evaluation and, therefore, are not reflected by the P200 [105].

Regarding the N200 component, considered to reflect an index of cognitive control, we hypothesized that BFRB and OCD groups would show a more significant N200 oddball effect, whereas the TS group would be comparable to controls. The N200 component showed a more prominent oddball effect over the right hemisphere at the fronto-central region. This anterior N200 oddball effect was not statistically significant in the BFRB and TS groups, contrary to the controls and OCD groups, which showed a significant frontal oddball effect. However, like the P200, with the addition of depression or anxiety as covariates, these group discrepancies disappear, which denotes that anxiety and depression tend to decrease or normalize slightly any group effect on these early amplitudes. This early effect pattern could suggest no difference in the processing related to the N200 (attention orienting).

Finally, the P300 component showed the most intricate and interesting finding. We hypothesized that the TS and BFRB groups would show a reduced oddball effect, while the opposite would be observed for the OCD group. The TS group showed a reduced P300 oddball effect, specifically in the frontal region, but no difference was detected for the BFRB group compared to the controls. The study by Morand-Beaulieu et al. [27] also showed a relatively intact amplitude of the P300 when using a visual motor oddball task. It is, therefore, reasonable to propose that the presence of BFRB does not reveal any anomaly in the electrocortical distribution differences related to stimulus evaluation processes reflected by the P300.

Regarding TS, some reported intact amplitude [30,32,36,37], while others observed a reduced amplitude [33] or an increase in P300 [43]. Despite these opposite results, some factors could explain these discrepancies. For instance, a small sample size or differences in the methodology used across studies, like the version of the oddball task (i.e., passive or active, visual or auditory), could be responsible for the pattern of results. However, we propose that the main factor that could explain these variable results might be due to the inclusion of TS participants who also showed OCD symptomatology. In that sense, the study of Thibault et al. [43] compared a relatively “pure” TS group and a “pure” OCD group to a TS+OCD group. Their results showed a larger P300 amplitude for the TS group in the rare condition, whereas the OCD and the TS+OCD group showed a reduced P300 amplitude. These results suggest that the addition of comorbid obsessive compulsive symptomatology to TS may attenuate the P300 component, even generating an activation pattern opposite to the one observed in the case of TS alone. In addition, another factor that could partly impact our P300 results remains the presence of anxious/depressive symptomatology. It seems that depressive and anxiety symptoms have an attenuating effect on the amplitude of the P300, as revealed by our covariance analyses. Consistently, several studies have revealed a decrease in this component for patients with depression [106,107,108,109].

However, our reduced P300 oddball effect derives mainly from increased frontal P300 amplitude in response to the frequent condition. A larger frontal P300 was noted in an earlier study but in the target condition [43]. The severity of symptoms in the TS group could explain these differences from that study. Indeed, while their group presents a moderate severity score of 22 on the Tourette Syndrome Global Scale (TSGS), our TS group had a YGTSS score of 38. Our findings could suggest that higher severity could lead to different alterations in the frontal P300 component. Given these results, we can posit that the implication of the dorsolateral prefrontal cortex and noradrenergic system underlies a larger P300 amplitude in the TS group in rare and frequent conditions. This P300 amplitude could reflect a higher state of arousal, which narrows the amount of attention available to modulate task performance in a typical oddball task requirement [110,111,112].

Unexpectedly, the OCD group also showed a comparable frontal P300 oddball effect to the TS group. However, these effects were obtained from two different patterns. Whereas the TS group showed greater frontal P300 amplitude in the frequent condition, the OCD group showed reduced frontal P300 amplitude in the rare condition, which corroborates some previous results in studies that used an oddball task [43,48,49,50,113,114]. That observation may be explained by an intolerance to uncertainty that mainly characterizes OCD. Therefore, participants may doubt their task performance [115,116], mainly during rare stimuli categorization.

It may be uneasy to reconcile these two theories, given that many studies report opposite results. As proposed in a review, the differences noted across studies may be explained by the severity of OCD symptoms, where greater severity is linked to a decrease in the amplitude of the P300 [117], which could feature the impact of OCD symptoms on processes mediated through the prefrontal cortical regions, which are hypothesized to be involved in memory inhibition mechanisms [118]. Moreover, Thibault et al. [43] observed that a higher severity level correlates with a more significant decrease in the P300 component. Thus, it is plausible that the decrease in P300 is caused by greater symptom severity, whereas the increase in P300 is observed more in patients with less severe symptoms [117].

OCD is composed of a wide variability of distinct profiles. Therefore, we propose that it is also plausible that the type of obsessions and compulsions expressed could explain this disparity across studies. For instance, obsessions with persistent doubts could instigate a decrease in P300, as described above, while obsessions linked to harm avoidance would, conversely, cause an increase. In that sense, studies that observed a larger P300 in OCD suggest this phenomenon may be explained by excessive demands on attentional resources for task-irrelevant stimuli [47,70,119,120]. Therefore, in terms of the disorder phenomenology, we suggest that this could be attributed to hypervigilance and excessive attentional focus on inoffensive external stimuli for people with harm avoidance obsessions. However, to our knowledge, none of the studies investigating the P300 component in OCD included subgroups divided by the symptoms’ themes, which prevents this hypothesis from being verified.

The divergence between the methodologies of these studies should not be overlooked, particularly the use of different paradigms to elicit the P300 since, as we know, evoked potentials can vary depending on the task demand [28]. In conclusion, it would be relevant for future studies to compare different levels of OCD severity and separate groups according to the nature of symptoms to see if different patterns of activation emerge for this component.

Finally, concerning the comorbidities, we observed that adding BAI scores did not affect the P300 amplitudes. However, when controlling for BDI scores, we no longer observed intergroup differences, despite the Condition by Region by Group interaction remaining close to the 0.05 threshold. Thus, anxious symptoms seem to not affect the P300 amplitude, unlike depressive symptoms, which seem to have attenuating effects. In that sense, several studies have revealed a decrease in this component for patients with depression [106,107,108,109]. Therefore, despite our results pointing toward a trend of alterations in P300 amplitude for our clinical groups when controlling for comorbidities, interpretation of these must be made with caution, given the presence of depressive symptomatology in our sample. It would, therefore, be interesting for future studies to investigate differences in P300 between patients with TS, OCD, and BFRB without comorbidities and patients with these disorders who also have anxiety and depressive symptoms. Nevertheless, it is essential to note that our initial results before covarying with BDI scores corroborate those of other studies that have controlled for depressive symptoms [43] or those that have excluded any participants with comorbidities [48,50], where P300 alterations were still observed for TS and OCD groups.

### 4.1. Source Localization

Because the most interesting results were related to the P300 oddball effect, we investigated whether the differences between groups originated from the same P300 sources or different regions. First, the most significant difference between the two conditions in the control group was in a 384–402 ms post-stimulus time frame. The source localization was estimated to be in the posterior cingulate cortex (BA30) and in the precuneus (BA31), which is localized in the parietal lobe (Table 2). The activation of these regions is in line with previous studies showing their implication in the oddball effect [121,122,123,124,125].

We observed the most significant difference between frequent and rare conditions for the TS group from 376 to 396 ms. No significant activation was obtained, meaning no region is more activated significantly for the P300 generation in the rare condition compared to the frequent condition despite a trend toward activation in the posterior cingulate cortex (BA23), like the control group. Therefore, we can suggest that even if differences were observed at the P300 scalp distribution, the generators appear comparable between those two groups but at a reduced strength for the TS group. It is important to note that previous studies reported anatomical and functional alterations of the cingulate cortex in TS [126,127], which could be consistent with our results. Previous results obtained with a motor oddball revealed that generators are more significant in the superior and middle frontal gyri (BA10), paracentral lobule (BA31), and cingulate gyrus (BA24) [38]. Thus, the methodology could explain the disparity between our results and theirs, suggesting that motor responses bring activations that affect the P300 amplitude distribution.

For the BFRB group, we observed activation in the cingulate gyrus, as we did for the control group, which is in line with the fact that no group difference was observed regarding the amplitude of the P300 between these two groups. These results suggest that the BFRB group is comparable to the control group regarding the cognitive functions reflected by the P300 and the associated brain regions. Thus, similar activation patterns to those of the control group are observed for both disorders. We think it is plausible that the cognitive task used in our study does not highlight the deficits known to be present in TS and BFRB. Indeed, as mentioned in the introduction, these two disorders are mainly characterized by impulses pushing people to produce gestures to reduce internal tension.

Moreover, difficulties in inhibiting these behaviors seem to be an essential feature of TS psychopathology and BFRB. Thus, we believe a task soliciting a motor response and inhibition capacities would highlight these difficulties. Only one study has conducted source-location analysis for the P300 oddball effect of these two disorders, where a motor task was used [38]. This paradigm required participants to inhibit their preprogrammed motor response associated with the frequent stimulus to produce another response when the rare stimulus appeared. These authors noticed a decreased P300 oddball effect for the BFRB group and generators in the inferior parietal lobule, postcentral gyrus, and superior temporal gyrus. They suggested that the decrease in amplitude observed for the BFRB group might reflect difficulties in refocusing attention on the task since these participants are overwhelmed by urges to produce their habits. Along with our results, we could add that the group differences observed in the generating source between BFRB and controls would be more salient during a motor than a non-motor oddball task.

Regarding the OCD group, the maximum difference between the two conditions was observed at around 400 ms post-stimulus. The regions significantly activated were the cuneus, lingual gyrus, medium occipital gyrus, posterior cingulate gyrus, and precuneus. Although some of these regions were also activated to generate the P300 oddball effect in the control group, these activations appear more prominent in the OCD group as other regions were also significant. The lingual gyrus was previously shown to be involved in processing emotional stimuli [128,129,130,131]. It has been suggested that this region might be involved in the phenomenology of OCD in a way that intrusive thoughts toward visual stimuli create negative emotions, such as in the case of obsessions of contamination [132]. In addition, neuroimaging studies have revealed alterations of this region in OCD, i.e., reduced connectivity of white matter fibers [133], reduced functional connectivity at resting state [134] as well as overactivation in a decision-making task and a positive correlation between this overactivation and symptom severity [135]. In short, the activation of this region in the generation of the P300 oddball effect in our OCD group could be explained by the presence of intrusive thoughts eliciting negative emotions during the experiment. The middle occipital gyrus has previously been shown to be involved in the pathophysiology of OCD. A correlation between glutamate levels in the thalamus and functional connectivity of the medium occipital gyrus has been observed [136]. Studies have noted the involvement of the glutaminergic neurotransmission system in OCD [137,138]. In addition, one study showed an increase in the gray matter volume of the middle occipital gyrus in the OCD group [139]. Thus, these observations point toward alterations in this region, which could justify its involvement in generating the oddball effect of OCD differently from the control group.

Finally, our results do not corroborate those of Andreou et al. [70], who also used the sLORETA method to localize P300 generators in OCD. Nevertheless, whether these authors proceeded to localize generators on the difference between the rare and frequent conditions is not specified, which is the case in our study. Instead, they report contrasting the OCD group with the control group, but no information is given as to whether this was done on the oddball effect or one condition in particular. It is, therefore, difficult to compare these results with our own. A recent study also used this method to locate sources of P300 in the OCD population, but this was carried out only on the rare stimuli of a motor auditory oddball task [140]. The results showed no difference in P300 activation across groups, both at the cortical level and at the level of generator sources. Once again, since our sLORETA results were obtained due to the difference between the two conditions for each group, the comparison between them and the ones in Flasbek et al. [140] remains limited.

### 4.2. Limits and Future Research

Our study’s principal limit remains our groups’ relatively small sample sizes. Although the initial database was extensive, we excluded some participants because of EEG signal alterations or technical problems. Of course, the presence of comorbidities is an important factor in the study of evoked potentials in these clinical populations, particularly in the case of anxiety and depressive symptoms. Indeed, the addition of anxiety and depression severity scores as covariables affected the amplitude of the components. Nevertheless, as mentioned in the introduction, a significant proportion of these clinical populations also suffer from other comorbid disorders [12,18,20]. Thus, despite the inclusion of participants suffering from anxiety and depression in our sample, it can be said that it is more representative of the general population than if we had only included participants with “pure” TS, OCD, or BFRB. However, it would be interesting to further investigate the effects of comorbidities on these clinical populations’ evoked potentials by integrating patients with and without concomitant disorders. Finally, another limit is that we did not distinguish different levels of symptom severity and their potential effects on the components’ amplitude. It might be interesting for future studies to incorporate broader levels of severity of tics, habits, and obsessive compulsive symptoms to investigate if different patterns of alterations emerge and to apply a more robust correlation between symptom severity and brain activity.

## 5. Conclusions

To our knowledge, this is the first study comparing TS, OCD, and BFRB. Our results allowed for a better comprehension of the group electrocortical differences in time (when) and location (where), which supports some of the previous results. No differences were observed for the N200 and P200 components when controlling for anxious and depressive symptoms, whereas the P300 oddball effect was impacted in specific groups. To sum up, our results showed that TS and OCD might show alterations in cognitive processes as reflected by a reduced P300 oddball effect compared to the control and BFRB groups. To an extent, that effect seemed to originate from different brain regions of P300 activation for the OCD group. It seems that both TS and OCD groups share deficits in anterior P300 activation but reflect distinct brain-generating source activations.

## Figures and Tables

**Table 1:** Clinical and sociodemographic data. Means (standard deviation).

	TS (*n* = 24)	BFRB (*n* = 16)	OCD (*n* = 18)	Controls (*n* = 59)	ANOVA
Age (years)	37 (11)	41 (15)	43 (15)	38 (12)	ns
Sex (M/F)	16/8	3/13	11/7	41/18	BFRB vs. TS (*p* = 0.009) BFRB vs. OCD (*p* = 0.048) BFRB vs. Controls (*p* = 0.001)
Intelligence (Raven’s Progressive Matrices)	82 (22)	81 (16)	74 (29)	82 (19)	ns
Laterality	19/2	12/2	16/2	58/0/1	ns
Anxiety (BAI)	9 (6)	10.1 (7)	15 (12)	3.9 (4)	Controls vs. TS (*p* = 0.004) Controls vs. BFRB (*p* = 0.017) Controls vs. OCD (*p* = 0.004)
Depression (BDI)	14 (11)	17 (9)	17 (8)	3 (4)	Controls vs. TS (*p* ≤ 0.001) Controls vs. BFRB (*p* ≤ 0.001) Controls vs. OCD (*p* ≤ 0.001)
Tics severity (YGTSS)	38 (17)	28 (8)	-	-	T = 2.52 (*p* = 0.017)
OCD symptoms’ severity (YBOCS)	-	-	27 (6)	-	

TS: Tourette Syndrome; BFRB: Body-focused Repetitive Behaviors; OCD: Obsessional-compulsive disorder; Laterality: Edinburgh Handedness Inventory; BAI: Beck Anxiety Inventory; BDI: Beck Depression Inventory; YGTSS: Yale Global Tic Severity Scale; YBOCS: Yale-Brown Obsessive Compulsive Scale; ns: non-significant. Significant results are defined by a threshold of ≤0.05.

**Table 2:** Coordinates of the significant areas generating the oddball effect in the control group.

Lobe	Structure	Brodmann Area	Coordinates						Voxel Value
			MNI			Talairach			
			X	Y	Z	X	Y	Z	
Limbic	Posterior cingulate cortex	23	−5	−30	25	−5	−28	24	1.03794
Limbic	Cingulate gyrus	23	0	−35	25	0	−33	25	1.03262
Limbic	Posterior cingulate cortex	30	5	−45	20	5	−43	21	1.03107
Limbic	Cingulate gyrus	31	0	−40	25	0	−38	25	1.01665
Limbic	Posterior cingulate cortex	29	10	−45	5	10	−43	7	9.31017
Limbic	Parahippocampal gyrus	27	10	−35	0	10	−34	2	9.15933
Parietal	Precuneus	31	10	−50	30	10	−47	30	9.08931
Limbic	Parahippocampal gyrus	30	10	−40	0	10	−39	2	9.08438

Talairach/MNI (Montreal Neurological Institute) coordinates and the significant value (<0.05) of the activated voxel.

**Table 3:** Coordinates of significant areas generating the oddball effect in the OCD group.

Lobe	Structure	Brodmann Area	Coordinates						Voxel Value
			MNI			Talairach			
			X	Y	Z	X	Y	Z	
Occipital	Cuneus	18	−25	−95	−5	−25	−92	0	1.33097
Occipital	Lingual gyrus	18	−20	−100	−10	−20	−97	−4	1.33039
Occipital	Lingual gyrus	17	−20	−95	−5	−20	−92	0	1.31363
Occipital	Medim occipital gyrus	18	−25	−95	0	−25	−92	5	1.30333
Occipital	Medium occipital gyrus	19	−25	−90	5	−25	−87	9	1.30078
Limbic	Posterior cingulate cortex	31	−5	−55	20	−5	−52	21	1.29951
Parietal	Precuneus	31	−5	−50	30	−5	−47	30	1.28793
Limbic	Posterior cingulate cortex	23	0	−50	25	0	−47	25	1.28386
Occipital	Inferior occipital gyrus	18	−30	−95	−10	−30	−92	−4	1.27584
Limbic	Cingulate gyrus	31	0	−55	25	0	−52	26	1.27503
Occipital	Cuneus	17	−20	−85	5	−20	−82	9	1.26789
Limbic	Posterior cingulate cortex	30	−5	−50	20	−5	−48	21	1.25667
Occipital	Inferior occipital gyrus	17	−20	−95	−15	−20	−93	−8	1.25145

Talairach/MNI (Montreal Neurological Institute) coordinates and the significant value (<0.05) of the activated voxel.

## Data Availability

The anonymized data presented in this study are available at the request of the corresponding author in conformity with our local ethics committee guidelines.
